# Homogeneous batch micro-crystallization of proteins from ammonium sulfate

**DOI:** 10.1107/S2059798320015454

**Published:** 2021-01-26

**Authors:** Claudia Stohrer, Sam Horrell, Susanne Meier, Marta Sans, David von Stetten, Michael Hough, Adrian Goldman, Diana C. F. Monteiro, Arwen R. Pearson

**Affiliations:** a Biomedical Sciences, University of Leeds, Woodhouse Lane, Leeds LS2 9JT, United Kingdom; b Hamburg Centre for Ultrafast Imaging, Institute for Nanostructure and Solid State Physics, Universität Hamburg, CFEL, Building 99, Luruper Chaussee 149, 22761 Hamburg, Germany; c European Molecular Biology Laboratory (EMBL), Hamburg Unit c/o DESY, Notkestrasse 85, 22607 Hamburg, Germany; d School of Life Sciences, University of Essex, Wivenhoe Park, Colchester CO4 3SQ, United Kingdom; e Biological and Environmental Sciences, University of Helsinki, Viikinkaari 5, FIN-00014 Helsinki, Finland; f Hauptman–Woodward Medical Research Institute, 700 Ellicott Street, Buffalo, NY 14203, USA

**Keywords:** microcrystals, batch crystallization, serial crystallography, ammonium sulfate

## Abstract

The emergence of X-ray free-electron lasers has led to the development of serial macromolecular crystallography techniques, making it possible to study smaller and more challenging crystal systems and to perform time-resolved studies on fast time scales. For most of these studies the desired crystal size is limited to a few micrometres, and the generation of large amounts of nanocrystals or microcrystals of defined size has become a bottleneck for the wider implementation of these techniques. Despite this, methods to reliably generate microcrystals and fine-tune their size have been poorly explored. Working with three different enzymes, l-aspartate α-decarboxylase, copper nitrite reductase and copper amine oxidase, the precipitating properties of ammonium sulfate were exploited to quickly transition from known vapour-diffusion conditions to reproducible, large-scale batch crystallization, circumventing the tedious determination of phase diagrams. Furthermore, the specific ammonium sulfate concentration was used to fine-tune the crystal size and size distribution. Ammonium sulfate is a common precipitant in protein crystallography, making these findings applicable to many crystallization systems to facilitate the production of large amounts of microcrystals for serial macromolecular crystallography experiments.

## Abstract 2

This work demonstrates how the precipitating properties of ammonium sulfate can be exploited to drive the transition from vapour-diffusion conditions to large-scale batch micro-crystallization and how the specific ammonium sulfate concentration can further be used to fine-tune microcrystal size and size distribution.

## 1. Introduction   

X-ray crystallography is still the most prevalent technique for solving the structures of soluble proteins. Conventionally, this requires a large, well ordered, single crystal in order to collect a complete data set by rotating the crystal through the X-ray beam. However, the use of serial crystallography approaches [serial synchrotron crystallography (SSX) or serial femto­second crystallography (SFX)] at X-ray free-electron lasers (XFELs) and third- and fourth-generation synchrotron sources has shifted the focus onto using smaller and smaller crystals (Chapman *et al.*, 2011 ▸; Neutze *et al.*, 2000 ▸; Martin-Garcia *et al.*, 2017 ▸). Although the production of large amounts of microcrystals is not yet routine, the serial data-collection approach has a number of advantages over single-crystal rotation crystallography, for example, the structure solution of more challenging proteins, such as integral membrane proteins, where only small crystals can be obtained (Liu *et al.*, 2013 ▸). In addition, as serial approaches are based on the collection of single diffraction patterns from many crystals and merging these still images to give a complete data set, this method allows room-temperature (RT) data collection to be performed with limited radiation damage (Suga *et al.*, 2015 ▸; Weinert *et al.*, 2017 ▸; Ebrahim, Moreno-Chicano *et al.*, 2019 ▸). The other major advantage is the pursuit of time-resolved (TR) studies (Šrajer & Schmidt, 2017 ▸; Levantino *et al.*, 2015 ▸; Neutze & Moffat, 2012 ▸). For time-resolved experiments, the crystal size is limited by the need for the uniform activation of reactions (for example by laser or substrate diffusion) across the whole crystal. For laser activation, depending on the absorption coefficient of the chromophore, the maximal crystal thickness is ∼20 µm (Levantino *et al.*, 2015 ▸). For diffusion-based reaction-initiation methods, even crystals as thick as 20 µm can still allow TR studies of slow reaction steps of the order of 10–100 ms. It is important to note that the time-resolution defined by diffusion is dependent on the thinnest crystal dimension (Mehrabi *et al.*, 2019 ▸; Schmidt, 2013 ▸; Makinen & Fink, 1977 ▸). This means that the generation of suitable nanocrystals or microcrystals of defined size can be a significant bottleneck for the wider implementation of these techniques.

To allow easy handling and delivery of crystals to the X-ray beam, as well as for synchronized protein activation for time-resolved studies, crystal size should ideally be tuneable and monodisperse. The desired crystal size will depend on the experimental setup used. For microfluidics experiments (liquid jets and microfluidic chips), crystal size is limited by the size of the nozzle or the channel of the delivery system (for a recent review of sample-delivery systems and their requirements, see Cheng, 2020 ▸). Clogging and jet instability can be significant issues when working with liquid jets, causing substantial delays and reducing the hit rate, and both of these issues are greatly aggravated by polydisperse crystal size distributions (Ibrahim *et al.*, 2015 ▸), as well as evaporative cooling, which causes the carrier solution to dry or freeze (Martiel *et al.*, 2019 ▸). Large variations in crystal size will also lead to variation in the measured intensities, making it difficult to tune the exposure time necessary to obtain good diffraction without the appearance of overloads, and posing a challenge for good scaling and merging of data (White *et al.*, 2012 ▸).

Many time-resolved experiments pursue photoactivatable reactions, which are typically initiated by a short laser pulse followed by a probe [recording of X-ray image(s)] after a defined delay time (Jung *et al.*, 2013 ▸; Schotte *et al.*, 2003 ▸; Tenboer *et al.*, 2014 ▸; Kupitz, Basu *et al.*, 2014 ▸; Barends *et al.*, 2015 ▸). Homogeneous activation using a laser requires an appropriate laser penetration depth relative to the size of the crystal under investigation (Levantino *et al.*, 2015 ▸; Grünbein *et al.*, 2020 ▸). Owing to absorption of the pumping laser light, larger crystal sizes result in nonhomogeneous activation throughout the crystal and thus a smaller population of active species in the X-ray-illuminated volume. This leads to a reduced signal from the desired activated state relative to the inactive state, making the interpretation of structural changes more difficult. A second protein-activation approach employs the delivery of substrates or ligands by diffusion (Mehrabi *et al.*, 2019 ▸; Beyerlein *et al.*, 2017 ▸; Olmos *et al.*, 2018 ▸). In addition to other factors, such as the diffusion coefficient of the ligand, rapid mixing of a ligand is dependent on the diffusion length of the crystal (Schmidt, 2013 ▸), and varying crystal sizes will lead to a blurring of mixing times and therefore of the time-resolution due to uncertain diffusion times.

In order to obtain a more homogeneous crystal sample, filtering can be performed to remove large crystals from slurries of smaller crystals. However, this becomes very in­efficient with a continuous size distribution. Furthermore, filtering can also cause undue shear stress and mechanical damage that leads to high mosaic spread and streaking of spots, potentially leading to complications during indexing (Dods *et al.*, 2017 ▸).

Recent methods development for SSX/SFX has focused heavily on sample delivery and has provided a number of viable methods for data collection (Martiel *et al.*, 2019 ▸; Grünbein & Kovacs, 2019 ▸; Sui & Perry, 2017 ▸; Cheng, 2020 ▸; Monteiro *et al.*, 2019 ▸, 2020 ▸; Mehrabi *et al.*, 2020 ▸). However, many of these techniques are now running into problems with sample consumption/availability when applied to more challenging samples (Cheng, 2020 ▸). This problem is amplified when considering a time-resolved experiment, where multiple complete data sets need to be collected to cover the time points of interest.

While methods to produce high-quality microcrystals and nanocrystals are highly desired, methods development in this area is still in its infancy. Several different strategies have been reported to create microcrystals, employing free-interface diffusion (Kupitz, Grotjohann *et al.*, 2014 ▸), controlled supersaturation (Lee *et al.*, 2018 ▸) and microseeding (Ibrahim *et al.*, 2015 ▸; Dods *et al.*, 2017 ▸). Unfortunately, many of these protocols do not suggest how to scale these conditions to volumes applicable to SSX/SFX.

Batch crystallization is one of the most frequently used strategies to produce high-density suspensions of microcrystals, as it is usually easier to scale up to the large volumes required compared with vapour diffusion. Furthermore, this technique allows the crystal density to be adjusted as needed for the SSX/SFX experiment, as crystals can be concentrated by low-speed centrifugation. To grow crystals in batch, the protein is mixed directly with a precipitant solution, aiming to place the protein within the nucleation zone after mixing (Russo Krauss *et al.*, 2013 ▸). Batch crystallization is conventionally approached by first determining a crystallization phase diagram for the protein of interest. To this end, the solubility limit of the protein can be determined by stepwise dissolution of crystals, by small-scale batch experiments with added seed crystals (Kupitz, Grotjohann *et al.*, 2014 ▸) or by careful experimental mapping of the crystallization space by varying the protein and precipitant concentrations (Beale *et al.*, 2019 ▸). However, this can be tedious and time-consuming (requiring several steps of optimization) and is not always repeatable between protein batches or days due to environmental factors in the laboratory such as temperature and relative humidity.

This work aims to provide a guide for the quick transition from known vapour-diffusion conditions to large-scale batch crystallization. To circumvent the tedious determination of phase diagrams, we take advantage of the excellent precipitation properties of ammonium sulfate. Ammonium sulfate is commonly used to precipitate proteins as both of its ions have a great ability to ‘salt out’ proteins (Hofmeister, 1888 ▸). It is thus not surprising that ammonium sulfate is also a successful precipitant for protein crystallization (Gilliland *et al.*, 1996 ▸; Gilliland, 1988 ▸). Of the 37 329 unique protein crystal structures deposited in the PDB^1^ that provide information about the crystallization conditions used, 10.1% used ammonium sulfate during crystallization. In 7.3% of the 37 329 conditions ammonium sulfate is probably acting as the precipitant rather than as a salt additive, as its concentration is higher than the cutoff reported previously by Beale *et al.* (2019 ▸).

Salt-based crystallization systems can also be of great advantage for serial experiments using sample-delivery systems in which the high viscosity of the crystal slurries, owing to the PEGs used for crystallization, is a limiting factor, for example for acoustic drop ejection (Davy *et al.*, 2019 ▸).

Working with three different soluble enzymes, *Escherichia coli* l-aspartate α-decarboxylase (ADC; a 60 kDa homotetramer), *Achromobacter cycloclastes* copper nitrite reductase (AcNiR; a 120 kDa homotrimer) and *E. coli* copper amine oxidase (ECAO; a 165 kDa homodimer), we show that when using ammonium sulfate the complexity of a crystallization experiment can be simplified by assuming that crystallization is mainly driven by the precipitant. We could therefore use increasing concentrations of ammonium sulfate as a means of generating microcrystals in a batch. We further explored how crystal size and size distribution can be fine-tuned in order to easily generate large amounts of monodisperse microcrystals for SSX/SFX experiments.

While most of the existing protocols for the generation of small crystals focus on very small crystals (<10 µm) for SFX experiments (Dods *et al.*, 2017 ▸; Ibrahim *et al.*, 2015 ▸; Beale *et al.*, 2019 ▸; Kupitz, Grotjohann *et al.*, 2014 ▸; Lee *et al.*, 2018 ▸; Wu *et al.*, 2015 ▸), SSX experiments can benefit from slightly larger crystals (∼20–50 µm) owing to the reduced beam intensity available (Martin-Garcia *et al.*, 2017 ▸). It is thus of great interest to be able to adjust crystal size according to the planned experiment, rather than to have to adjust the experiment according to the crystals available.

Here, we demonstrate for these three proteins that exploiting the precipitating properties of ammonium sulfate provides a straightforward approach to transition from vapour diffusion to batch and can even provide a way to adjust crystal size. As ammonium sulfate is a common precipitating agent, this approach should be applicable to other proteins where crystallization conditions that include ammonium sulfate are known.

## 2. Methods   

### 2.1. Preparation of crystallization solutions   

All crystallization solutions were prepared by dissolving the powders or liquids of the corresponding salts and buffer components (from Sigma–Aldrich or Carl Roth) and making up to the total volume with double-distilled water. The pH was adjusted following the corresponding acid–base pair. All solutions were filtered using 0.1 µm syringe filters.

### 2.2. Protein-purification protocols   

#### 2.2.1. Purification of ADC   

ADC was purified as described previously (Monteiro *et al.*, 2015 ▸; Arnott *et al.*, 2017 ▸). In brief, ADC was overexpressed from the vector pRSETA-ADC-WT (Saldanha *et al.*, 2001 ▸) in *E. coli* BL21 (DE3) cells using an auto-induction protocol under ampicillin selection (100 µg ml^−1^) at 37°C for 16 h (Studier, 2005 ▸). The cells were collected by centrifugation (30 000*g*, 45 min), resuspended in 50 m*M* K_2_HPO_4_ pH 7.4, 300 m*M* NaCl, 10 m*M* imidazole and mechanically disrupted. The protein was purified by Ni–NTA affinity using a 5 ml HisTrap pre-packed column (GE Healthcare) and eluting with 50 m*M* K_2_HPO_4_ pH 7.4, 300 m*M* NaCl, 250 m*M* imidazole. Fractions containing the pure protein were pooled, concentrated and buffer-exchanged into 50 m*M* Tris pH 7.5, 100 m*M* NaCl, 0.1 m*M* DTT using a 5 ml HiTrap desalting column (GE Healthcare). The final protein sample was concentrated to 25 mg ml^−1^ using a 10 kDa molecular-weight cutoff Millipore concentrator.

#### 2.2.2. Purification of ECAO   

ECAO protein was prepared from *E. coli* BL21 (DE3) cells carrying the recombinant plasmid pKKECAO (Murray *et al.*, 1999 ▸) according to previously reported procedures (Smith *et al.*, 2010 ▸) with a few modifications. Freshly transformed cells were grown in LB medium supplemented with 200 µg ml^−1^ ampicillin and 2 m*M* CuSO_4_ at 37°C to an OD_600_ of 0.7. Expression of ECAO was induced by the addition of isopropyl β-d-1-thiogalactopyranoside (IPTG) at a final concentration of 1 m*M* and protein was expressed overnight at 30°C. The cells were harvested and the cell pellet was resuspended in 50 m*M* Tris–HCl pH 8.0 containing 20%(*w*/*v*) sucrose and 2 m*M* CuSO_4_. Subsequently, lysozyme was added to the cells to a final concentration of 2.3 mg ml^−1^ and incubated for 1 h with gentle agitation. 8 m*M* EDTA and 5.7 m*M* MgCl_2_ were added under constant stirring and the sucrose concentration was diluted to 14%(*w*/*v*). The periplasmic fraction was then isolated by centrifugation for 30 min at 8000*g*. The supernatant was supplemented with protease inhibitors (one EDTA-free cOmplete tablet, Roche), dialysed overnight against 20 m*M* Tris–HCl pH 7.0 and subsequently purified by ion-exchange chromatography using a Q Sepharose column eluted with a gradient from 35 to 175 m*M* NaCl in 20 m*M* Tris–HCl pH 7.0. Protein-containing fractions were pooled, concentrated to a final volume of 5 ml and purified by size-exclusion chromatography (Superdex 200) in 20 m*M* Tris–HCl pH 7.0. ECAO-containing fractions were pooled, and excess CaCl_2_ and CuSO_4_ were removed by dialysis against 20 m*M* Tris–HCl pH 7.0 overnight. The dialysed solution was concentrated to 12 mg ml^−1^ using a 100 kDa molecular-weight cutoff Millipore concentrator.

#### 2.2.3. Purification of AcNiR   

AcNiR protein was prepared following a previously published protocol (Horrell *et al.*, 2016 ▸). In brief, a pET-26b plasmid containing the codon-optimized gene for AcNiR (GenScript) was transformed into *E. coli* BL21 (DE3) cells in the presence of 30 µg ml^−1^ kanamycin. LB cultures were grown with 2 m*M* CuSO_4_ to an OD_600_ of 0.4–0.6 and were induced with 2 m*M* IPTG at 18°C for 16 h. The cultures were harvested and the pellets were resuspended in 20 m*M* Tris–HCl pH 7.5, 150 m*M* NaCl. The cells were lysed by sonication for five cycles of 30 s on and 30 s off and clarified by centrifugation at 30 600*g* for 15 min at 4°C. The cleared lysate was dialysed for 3 h against 2 m*M* CuSO_4_, 20 m*M* Tris–HCl pH 7.5 at 4°C. Excess CuSO_4_ was removed by dialysis against 20 m*M* Tris–HCl pH 7.5, leaving a protein solution with a distinct green colour that is consistent with formation of the holoenzyme.

In a procedure adapted from Horrell *et al.* (2016 ▸), the cleared, dialysed lysate was loaded onto a gravity-flow hydroxyapatite column (Bio-Rad, Hercules, California, USA) pre-equilibrated in 20 m*M* Tris–HCl pH 7.5 and washed with 20 ml 20 m*M* Tris–HCl and 20 ml 10 m*M* K_2_HPO_4_. AcNiR was eluted with an increasing concentration of K_2_HPO_4_ from 10 to 150 m*M* by observing the green band of protein moving down the column. The eluted protein was concentrated and further purified by ammonium sulfate precipitation. 120 µl protein solution was added to 240 µl 20 m*M* Tris–HCl pH 7.5 and 40 µl 100 m*M* sodium acetate pH 4.75. 25 µl 4 *M* ammonium sulfate was then added to the protein solution and left for 10 min at room temperature (RT) before pelleting the precipitated protein by centrifugation at 16 900*g* for 10 min at RT. The supernatant was pipetted into a fresh Eppendorf tube and the process was repeated, progressively increasing the ammonium sulfate concentration, until the pellet appeared green. Excess ammonium sulfate was added to precipitate all of the remaining AcNiR protein, which was pelleted by centrifugation, resuspended in 0.5 ml 50 m*M* MES pH 6.5 and concentrated to 20 mg ml^−1^.

### 2.3. Crystallization in batch   

#### 2.3.1. Crystallization of ADC   

Buffer-exchanged ADC was concentrated to 25 mg ml^−1^ and mixed with precipitant solution (1.85–2.10 *M* ammonium sulfate, 65 m*M* citric acid, 71 m*M* Na_2_HPO_4_ pH 3.8) in a 1:3 protein:precipitant solution ratio. The solutions were mixed by vortexing for 10 s and were then incubated at 18°C for 1–2 days. Initial crystallization was carried out in 0.5 ml Eppendorf tubes, with a final volume of 80 µl. The process was then scaled up to 800 µl in 1.5 ml tubes. To further increase the monodispersity of crystal size and for the highest reproducibility, ammonium sulfate solutions were handled in a humidity-controlled environment, for example, by using a glove bag in which humidity was reduced by using Drierite (with indicator, 4 mesh; Acros Organics).

#### 2.3.2. Crystallization of ECAO   

Buffer-exchanged ECAO was concentrated to 12–36 mg ml^−1^. The protein was mixed with a precipitant solution consisting of 2.9–4.0 *M* ammonium sulfate, 0.1 *M* Tris pH 8.3–8.5 in a 1:3 protein:precipitant solution ratio with 1 µl of microseeds. The mixture was quickly vortexed and incubated at 18°C. To generate the microseeds, crystals grown by vapour diffusion (6–10 mg ml^−1^ protein, 1.1–1.3 *M* sodium citrate, 0.1 *M* Tris pH 6.8–7.3 reservoir solution) were disrupted using seed beads (Hampton Research).

#### 2.3.3. Crystallization of AcNiR   

An initial micro-crystallization protocol for AcNiR is given in Moreno-Chicano *et al.* (2019 ▸); the protocol reported here contains further optimization to increase the crystal quality and reproducibility. Purified AcNiR was concentrated to 20 mg ml^−1^. Precipitant solution (2.5 *M* ammonium sulfate, 0.1 *M* sodium citrate pH 4.75) was added in a 1:3 protein:precipitant solution ratio and mixed by repeatedly pipetting up and down. A milky green precipitate appeared immediately. To speed up the crystallization process and reduce the amount of large crystal aggregates, 2 µl of seeds were added following the mixing. Crystals appeared within the precipitate after incubation at RT for one day. To remove the precipitate, batches were centrifuged at 2300*g* for 1 min, leading to sedimentation of the crystals as a dark green band surrounded by precipitant. The precipitate was carefully removed with a pipette and the crystals were resuspended in 1.25 *M* ammonium sulfate, 50 m*M* sodium citrate pH 4.75. This process was repeated 1–2 times until most of the precipitate had been removed, as judged by inspection under a standard microscope.

### 2.4. Diffraction experiments   

#### 2.4.1. Oxford photochip   

Data from AcNiR microcrystals were collected at 12.65 keV using the Oxford photochip (Ebrahim, Moreno-Chicano *et al.*, 2019 ▸; Ebrahim, Appleby *et al.*, 2019 ▸) at the T-REXX endstation on the EMBL beamline P14 at the PETRA III synchrotron during a LAMA experiment (Mehrabi *et al.*, 2019 ▸). Microcrystals were mounted onto the chip as a slurry in crystallization mother liquor using the T-REXX Chip Loading Platform (Mehrabi *et al.*, 2020 ▸), and data were collected at room temperature. To obtain a sufficient number of indexed patterns, data collected from three chips were combined. Each of the chips contains 25 600 holes, but only a quarter of the holes (6400) were used for X-ray diffraction data collection to avoid contamination by droplets injected into neighbouring holes during the LAMA experiment. At each hole, 50 diffraction images (of 1.4 ms) were recorded in rapid succession. The substrate-containing droplet was injected onto the chip during the fourth image, and thus the first three diffraction images recorded from each hole represent the apo state of the protein. The 960 000 (= 3 × 50 × 6400 crystals) images were integrated with *CrystFEL* (White *et al.*, 2012 ▸) using *XGANDALF* (Gevorkov *et al.*, 2019 ▸) for indexing, which was able to successfully index 109 708 images. Afterwards, *ambigator* (White *et al.*, 2016 ▸) was used to resolve the *P*2_1_3 indexing ambiguity, and the data were scaled and merged into 50 individual data sets (corresponding to 50 time points from 1.4 to 70 ms) with *partialator* (White, 2014 ▸; White *et al.*, 2016 ▸). Only data from the first time point (*i.e.* before the injection of the substrate), obtained from 3018 indexed patterns, are presented here.

#### 2.4.2. 3D-MiXD device   

Data from ADC microcrystals were collected on beamline ID30A-3 (von Stetten *et al.*, 2020 ▸) at the ESRF using the 3D-MiXD microfluidic chip (the experiment is fully described in Monteiro *et al.*, 2020 ▸). Data were collected in flow using intermittent X-rays provided by the rotating fast shutter at 45–50% transmission and 5 ms exposure time, and were integrated and merged using *CrystFEL.*

Data from ECAO microcrystals were collected at room temperature using the 3D-MiXD chip at the T-REXX endstation on the EMBL beamline P14 at the PETRA III synchrotron at 12.65 keV with 5 ms exposure time. 95 527 images were recorded, and *CrystFEL* (using *iMOSFLM* for indexing; Battye *et al.*, 2011 ▸) was able to integrate 15 908 of these images, which were subsequently merged with *partialator* into a data set at 2.5 Å resolution.

## 3. Adjusting the ammonium sulfate concentration to navigate crystallization space   

To enable micro-crystallization in batch, the initial crystallization conditions must be inside the nucleation zone of the crystallization phase diagram (Rupp, 2015 ▸), and it is possible to estimate the required conditions for vapour-diffusion results as follows. Assuming full equilibration of a vapour-diffusion drop before crystallization, the maximal precipitant concentration approximately equals the precipitant concentration of the reservoir. Once equilibrium has been reached, the precipitant concentration remains constant. Reaching this point should occur more rapidly in salt-based crystallization conditions than in those where the precipitant is organic, such as polyethylene glycol. Thus, it is probable that we enter the nucleation zone around this precipitant concentration and we should aim for a similar precipitant concentration in the final batch condition. Usually, for batch crystallization, the protein and precipitant are mixed in a 1:1–1:3 ratio. Taking this dilution factor into account, the concentration of the precipitant for batch crystallization should be chosen to be 1.3–2 times higher than in the vapour-diffusion condition. As the protein solution is also diluted, the starting protein concentration also needs to be higher than for vapour diffusion. Surprisingly, for all three systems tested, the optimal final protein concentration in batch was lower than in the vapour-diffusion condition, suggesting that crystallization in ammonium sulfate conditions is mainly driven by the precipitant (Table 1 ▸).

The growth of protein crystals is mediated by slightly attractive protein–protein interactions (George *et al.*, 1997 ▸). An increasing ammonium sulfate concentration has been shown to drive protein–protein interactions towards a state favouring crystallization (Dumetz *et al.*, 2007 ▸). While this effect has been described for a variety of different proteins, the rate at which these attractive forces are established differs from case to case (Dumetz *et al.*, 2007 ▸). Small changes in ammonium sulfate concentration can have a strong effect on protein solubility and thus on the speed at which crystal nuclei are formed. Thus, fine-tuning the ammonium sulfate concentration can have beneficial effects. This can be performed either by adjusting the ammonium sulfate concentration directly, or indirectly by varying the protein:precipitant ratio.

### 3.1. Moving horizontally though the phase diagram (direct approach)   

**Figure 1:**
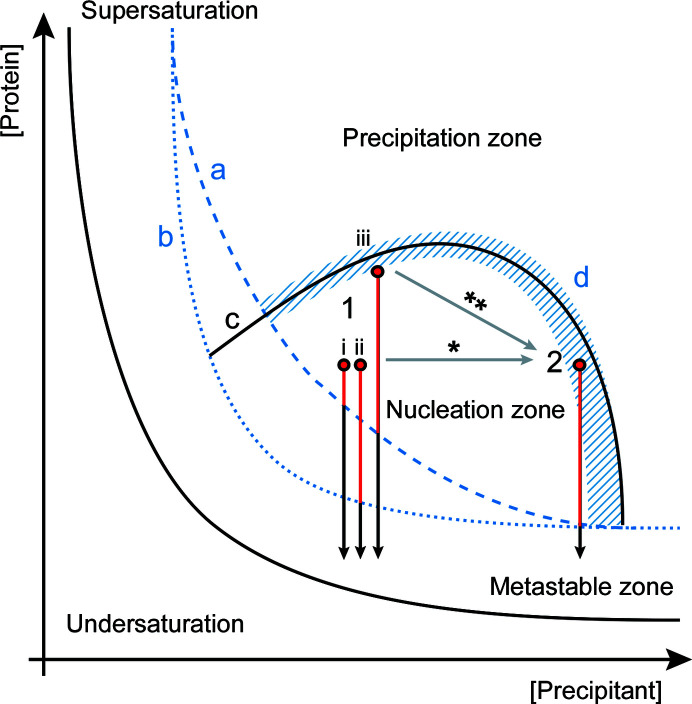
Schematic overlay of the crystallization phase diagrams for the three used model enzymes. The proposed boundaries between the metastable zone and the nucleation zone are drawn differently for ADC/AcNiR (dashed blue line a) and ECAO (dotted blue line b). The same generic boundary between the nucleation and the precipitation zone is drawn for ADC and ECAO (line c). AcNiR crystallizes from an initial precipitation phase; thus, there is no sharp boundary between the nucleation and precipitation zones, as indicated by the striped area in blue (labelled d). The grey arrows show the different ways of moving through the phase diagram. For ADC and ECAO the ammonium sulfate concentration was adjusted directly, resulting in a horizontal move along the phase diagram (*). For AcNiR this was performed by changing the protein:precipitant ratio, thereby moving on the diagonal (**). The vertical red/black arrows represent time spent in the metastable zone for batch crystallization at (1) a lower ammonium sulfate concentration for ADC (i), ECAO (ii) and AcNiR (iii) and (2) a higher concentration. The red part of the arrow illustrates the difference in time spent in the nucleation zone, depending on the starting point and the borderline between the metastable zone and the nucleation zone. For AcNiR no effect on crystal size could be observed, but effects on crystallization speed and the amount of accompanying amorphous precipitation were observed upon increasing the ammonium sulfate concentration.

The effects of changing the ammonium sulfate concentration will very much depend on the crystallization phase diagram of the investigated protein. However, even without knowing the specific phase diagram, but having a generic crystallization phase diagram in mind, there are only a few possible outcomes of increasing the ammonium sulfate concentration. Altering the ammonium sulfate concentration will cause us to move through the phase diagram parallel to the *x* axis. If we have a rather steep boundary between the metastable zone and the nucleation zone within the range of precipitant concentrations tested (Fig. 1 ▸, line a), at lower precipitant concentrations the starting point (1, i in Fig. 1 ▸) will be closer to the boundary and thus little time will be spent in the nucleation zone, leading to few, larger crystals. When increasing the precipitant concentration (2 in Fig. 1 ▸) more time will be spent in the nucleation zone, resulting in more and smaller crystals. However, if the boundary between the metastable and the nucleation zone is flatter (Fig. 1 ▸, line b), increasing the precipitant concentration (1, ii → 2 in Fig. 1 ▸) has less of an effect on the time spent in the nucleation zone and no effect on crystal size will be observed. Finally, if the nucleation zone is very narrow, increasing the precipitant concentration will quickly lead to precipitation.

**Figure 2:**
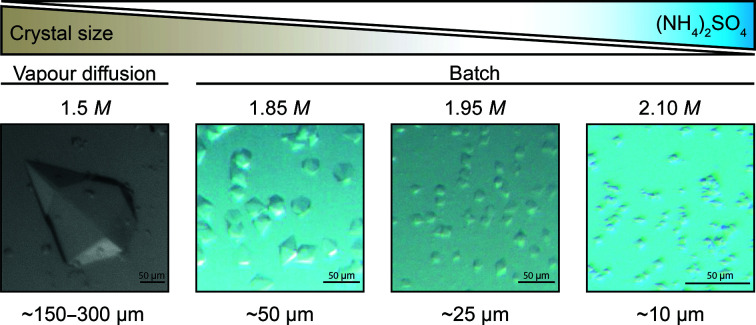
The crystal size of ADC is dependent on the ammonium sulfate concentration; increasing the concentration of ammonium sulfate leads to smaller crystals.

Even though an increase in ammonium sulfate concentration was required to go from vapour diffusion to batch micro-crystallization, the size of the ECAO microcrystals did not depend on different final ammonium sulfate concentrations between 2.2 and 3.0 *M*, thus implying a rather flat boundary in this range. This was not true for ADC, suggesting a rather steep boundary in the range of the tested conditions. Precipitant concentrations in the range 1.7–2.2 *M* ammonium sulfate usually gave crystals, while concentrations above this led to precipitation and concentrations below 1.7 *M* resulted in few, very large crystals. Controlling the exact precipitant concentration within this range led to highly tuneable crystal sizes. 2.1 *M* ammonium sulfate gave the smallest crystals (5–10 µm), which were suitable for SFX experiments, while 1.8 *M* ammonium sulfate gave 60 µm crystals suitable for SSX experiments (Fig. 2 ▸). Changes in ammonium sulfate concentration of as small as 50 m*M* were tested, which still led to considerable differences in crystal size. Because of the high sensitivity of the ADC crystallization system to the exact ammonium sulfate concentration, it was important to set up the crystallization experiments in a controlled environment, such as inside a glove bag, as the hygroscopic properties of ammonium sulfate cause changes in the concentration of the stock solutions relative to the local humidity levels, resulting in different concentrations in the final crystallization conditions and variations in crystal size (Supplementary Fig. S1). Similarly, protein age had a large impact on the crystallization reproducibility of ADC. When using protein that had been stored at 4°C for several weeks, the crystal size distribution was less homogeneous and some very large crystals appeared (Supplementary Fig. S1).

By adjusting the ammonium sulfate concentration, we were able to fine-tune the ADC crystal size according to the experimental requirements, enabling us to choose the experimental setup according to the scientific question rather than the properties of the crystals available. Precise control of precipitant concentration may thus be a generic method to control the size of the microcrystals for other proteins, as long as the precipitant has a similar phase diagram to that of ADC with ammonium sulfate.

### 3.2. Moving through the phase diagram on the diagonal (indirect approach)   

Another way of adjusting the precipitant concentration is by navigating though the crystallization space on the diagonal by changing the ratio of protein to precipitant rather than the precipitant concentration at a fixed protein:precipitant ratio (labelled ** in Fig. 1 ▸). This has the advantage that by decreasing the protein:precipitant ratio, the precipitant solution is less diluted and stays closer to the actual vapour-diffusion condition. This could explain why the best results were obtained for ADC when using a 1:3 protein:precipitant ratio while increasing the precipitant stock concentration ∼1.3 times. Nevertheless, testing different protein:precipitant ratios might help to further optimize crystallization conditions.

**Figure 3:**
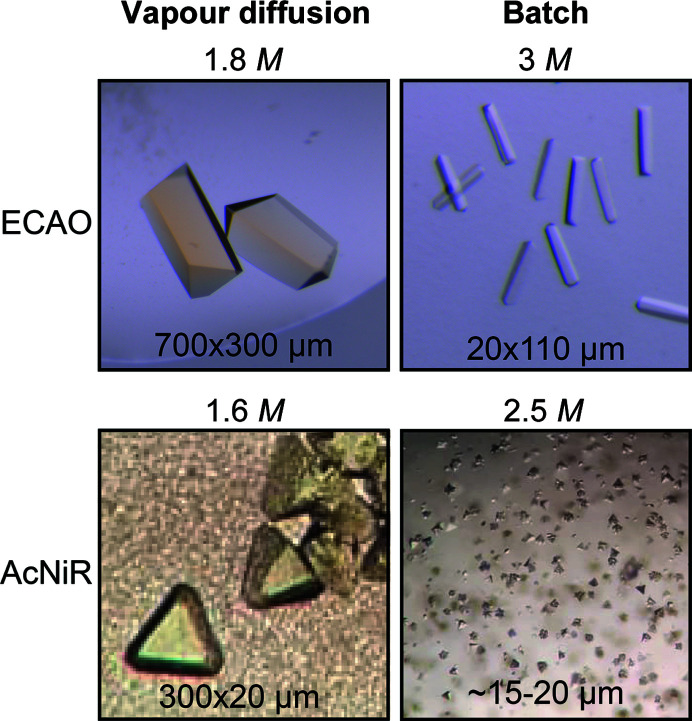
Comparison of ECAO (top) and AcNiR (bottom) crystals obtained by vapour diffusion (left) and batch micro-crystallization (right). For both proteins, obtaining microcrystals required an increase in the ammonium sulfate concentration (the concentration is indicated at the top of each image).

Unlike ADC and ECAO, AcNiR crystals emerged from an initial precipitation phase. The amount of precipitate formed was dependent on the protein:precipitant ratio and thus the final ammonium sulfate concentration (Fig. 3 ▸, bottom). The stock precipitant concentration was chosen in such a way that a 1:1 protein:precipitant ratio would result in a precipitant concentration slightly below vapour-diffusion conditions, a 1:2 ratio in the same concentration and a 1:3 ratio in a slightly higher concentration. Microcrystals formed under 1:3 and 1:2 protein:precipitant ratios and were accompanied by heavy and moderate precipitation, respectively. A 1:1 protein:precipitant ratio gave very light precipitation and no crystals were observed even weeks after mixing. At higher ammonium sulfate concentrations (1:3) the protein is driven out of solution more quickly and crystals appear more rapidly (after 3–4 days); however, this also leads to stronger amorphous precipitation. At a slightly lower precipitant concentration (1:2) the protein is driven out of solution more slowly, and consistently we only observed crystals to form after 4–5 days, but accompanied by less precipitation (Table 2 ▸). The undesired precipitate could be separated from the crystals during harvesting by several rounds of centrifugation, resulting in a more homogeneous final crystal solution (Supplementary Fig. S2). The initial precipitated phase is probably a mixture of amorphous aggregate and ordered nuclei required for crystallization. In such a case, the addition of crystal seeds might help to drive the equilibrium towards crystal formation, speeding up the process and making it more reproducible.

## 4. Other tools to increase crystal quality   

Batch crystallization of ADC and AcNiR only required adjustment of the ammonium sulfate concentration. ECAO, however, required further adjustments to produce microcrystals at all (Table 1 ▸).

### 4.1. Microseeding   

Seeding is commonly used to grow well ordered, large crystals; microseeding approaches can, however, also be used for the generation and optimization of microcrystals (Dods *et al.*, 2017 ▸; Ibrahim *et al.*, 2015 ▸). In microseeding, initial crystals are pulverized to microscopic particles and used to prepare a seed-stock mixture, providing the nuclei for crystallization (Luft & DeTitta, 1999 ▸). The number of crystals formed depends on the number of nucleation points; thus, more concentrated seed stocks will result in more, smaller crystals (D’Arcy *et al.*, 2003 ▸; Ibrahim *et al.*, 2015 ▸).

Two of our tested systems benefited from the addition of crystal seeds. To generate seeds, crystals grown by vapour diffusion were collected and crushed by vortexing using a seed tool kit (Hampton Research). Aiming to generate large amounts of small microcrystals, we added 1 or 2 µl undiluted seed stock to a batch crystallization mix of 80 or 800 µl in volume. Using an undiluted seed stock probably introduced such a high quantity of nucleation points that the effect of a slightly higher dilution by increasing the final batch volume did not lead to a significant change in crystal size and number within the volume range tested here. Therefore, for both proteins the same amount of seed stock could be used independently of the final volume, giving indistinguishable results.

**Figure 4:**
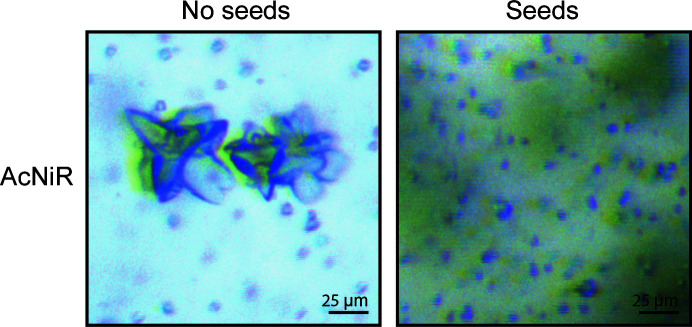
Microcrystals of AcNiR grown with and without the addition of seeds using a 1:3 protein:precipitant ratio. The addition of seeds led to a higher reproducibility and faster crystallization, as well as minimizing the formation of the larger clusters seen on the left. The pictures were taken before centrifugation to remove precipitate (green haze).

While ECAO crystals usually grow without seeding by vapour diffusion, microseeding was one of the key adjustments required for successful micro-crystallization in batch (Fig. 3 ▸, top), indicating that the conditions used were too mildly supersaturated to allow spontaneous nucleation. In contrast, AcNiR microcrystals were obtained without seeds in batch. However, adding seeds was still beneficial, as crystallization became more reproducible and resulted in fewer crystal aggregates (Fig. 4 ▸). It also significantly sped up the crystallization time from 3–4 days at a 1:3 protein:precipitant ratio to one day (Table 2 ▸).

### 4.2. Reducing the number of buffer and salt components   

In addition to salts, buffers are also weak electrolytes and therefore have electrophoretic effects in protein solutions. The adsorption of ions onto protein surfaces has been shown to play a key role in protein crystallization (Curtis & Lue, 2006 ▸). We therefore reasoned that reducing the complexity of our crystallization system as much as possible would increase its reproducibility.

The original vapour-diffusion condition of ECAO contained 100 m*M* sodium citrate as well as 100 m*M* Tris. The successful micro-crystallization of ECAO in batch required the omission of sodium citrate from the crystallization mixture (Table 1 ▸). This may be rationalized as Tris and sodium citrate have previously been reported to display opposite effects on electrophoretic protein mobility and in modulating the ion-specific properties, such as Hofmeister phenomena, of added salts (Cugia *et al.*, 2013 ▸). These effects are mainly caused by the adsorption of buffer molecules/ions at the protein surface, which will in turn affect protein crystallization (Cugia *et al.*, 2013 ▸; Curtis & Lue, 2006 ▸).

Modern-day crystallization conditions are often extremely complex, with exotic precipitants and the presence of multiple salts and additives. However, this was not always the case. In the early days of crystallography most proteins were crystallized from sodium chloride, ammonium sulfate, phosphate or, more recently, PEG, with grid screens testing pH versus precipitant concentrations. In more recent times (since the identification of 50 crystallization cocktails that gave a reasonable coverage of chemical space by Jancarik & Kim, 1991 ▸), most crystallization conditions have been optimized from an initial hit in a screen, where the buffers can be extremely complex. In our opinion, whilst using screens undoubtedly increases the number of initial hits that one might obtain for a protein, crystallizability is much more a function of protein sample quality than mother-liquor complexity, and in many cases simplified mother liquors may give crystals that are of as good quality and that are potentially more reproducible.

## 5. Upscaling   

Even though sample-delivery methods have advanced considerably to reduce sample consumption, most TR-SSX/SFX experiments still require a considerable amount of protein and are usually performed on protein targets that can be reliably produced in quantities of tens to hundreds of milligrams. Therefore, sample consumption is not typically the limiting factor when screening micro-crystallization conditions. By starting the transition to batch crystallization at the known conditions for vapour diffusion and increasing the ammonium sulfate concentration, the screening space is significantly reduced compared with the initial screening for crystal hits. We therefore screened batch conditions in relatively large volumes (80 µl) compared with, for example, microbatch. The great advantage of this is that scale-up should be possible without re-optimization. ADC was scaled up tenfold to 800 µl volumes without any further optimization step, and AcNiR was scaled up to a final volume of 2 ml. This is especially advantageous for time-resolved experiments, where many protein crystals with consistent properties are required. Larger batches also allow one to produce the amounts of microcrystals required quickly with minimal crystal handling, while avoiding problems generated by batch-to-batch variations, which could introduce polydispersity. Nonetheless, smaller reaction volumes of about 80 µl can be sufficient to generate the amounts of crystals required for a single serial data set quickly.

By following this approach and thereby avoiding any additional optimization steps, we managed to generate several millilitres of monodisperse ADC microcrystals within two weeks from initial hits.

## 6. Diffraction quality   

The diffraction quality of the crystals of all three proteins was tested at MASSIF3 (ID30-A-3, ESRF) and T-REXX (P14-EH2, PETRA III) and was shown to be comparable to that of single crystals obtained by vapour diffusion (Table 3 ▸). All proteins crystallized in the same space group as previously observed. While the single-crystal data sets for the large crystals of ECAO and ADC were collected at 100 K, all serial experiments using microcrystals were performed at RT.

Furthermore, the ADC crystals were used to test a new microfluidic device for TR-SSX data collection (Monteiro *et al.*, 2020 ▸) and the AcNiR microcrystals were used for data collection of protein–ligand complexes at RT on fixed targets for SFX (Moreno-Chicano *et al.*, 2019 ▸; Ebrahim, Appleby *et al.*, 2019 ▸).

## 7. Conclusions   

Here, we present a novel approach to produce large amounts of microcrystals quickly in batch starting from previously established vapour-diffusion conditions with ammonium sulfate. We successfully grew microcrystals of three protein systems using increased concentrations of ammonium sulfate without prior knowledge of the phase diagram beyond the reported vapour-diffusion crystallization conditions. We have demonstrated that the ammonium sulfate concentration can be used to control the crystal size and crystallization speed. The produced microcrystals were homogeneous in size, were readily produced in large batch volumes and diffracted similarly to the larger crystals, making them suitable for TR studies.

Serial crystallography represents the new frontier in X-ray crystallography and opens exciting opportunities for structural biology. Successful data collection depends on the supply of well diffracting microcrystals. To this end, a variety of delivery systems have been established, requiring different compositions of carrier media. For example, when using free jets [especially gas dynamic virtual nozzle (GDVN) systems], high salt concentrations can cause drying or freezing of the carrier solution, leading to clogging and intense background effects. Our micro-crystallization approach is based on adjusting the concentration of the precipitating agent ammonium sulfate, requiring rather high salt concentrations. However, when we tested the diffraction of our microcrystals with two different delivery systems (solid target and microfluidic chip), we did not experience any difficulties arising from the ammonium sulfate. Data from AcNiR microcrystals were collected using the Oxford photochip, which, as with many other chip-based approaches, combines crystal mounting with removal of the mother liquor, thereby reducing background issues caused by components from the carrier media. In contrast, when using the 3D-MiXD microfluidic chip, the crystals are delivered in their mother liquor. However, also in this setup, using slightly larger crystals on a synchrotron beamline, the observed background from the carrier solution was similar for ammonium sulfate and PEG/NaCl-based conditions (see Supplementary Fig. S2 in Monteiro *et al.*, 2020 ▸). In fact, when performing time-resolved experiments, for example using a 3D-MiXD microfluidic chip, the experiment might profit from a salt-based, and therefore low-viscosity, system that better facilitates diffusive mixing.

Besides ammonium sulfate, polyethylene glycols (PEGs) are the other most commonly used precipitant for protein crystallization. However, in contrast to ammonium sulfate and other salts from the Hofmeister series, the precipitating effect of PEGs is generally thought to work through attractive depletion and volume exclusion rather than electrolyte–non-electrolyte interactions as for ammonium sulfate (Asakura & Oosawa, 1958 ▸; Polson, 1977 ▸). PEGs have varying sizes and branching, which, together with the varying protein size and shape and local environmental conditions, have been shown to have a significant effect on their precipitating effect (Sim *et al.*, 2012 ▸). This suggests that PEG–protein interactions are more complex. Thus, the validity of applying our approach to PEG-driven crystallization remains to be empirically tested. Nonetheless, as ammonium sulfate is a common precipitation agent in protein crystallography, these findings will help to rationalize batch crystallization, facilitating the production of large amounts of microcrystals for SSX/SFX experiments where crystallization conditions that include ammonium sulfate are known.

## Supplementary Material

Supplementary Figures. DOI: 10.1107/S2059798320015454/nj5293sup1.pdf

## Figures and Tables

**Table 1:** Ammonium sulfate crystallization approaches: comparison of vapour-diffusion and batch crystallization conditions and the different factors aiding batch micro-crystallization of the three tested proteins ADC, ECAO and AcNiR

Protein	ADC	ECAO	AcNiR
Vapour-diffusion conditions	9–11 mg ml^−1^ protein, 1.5 *M* (NH_4_)_2_SO_4_, 0.1 *M* sodium citrate pH 3.8	6–10 mg ml^−1^ protein, 0.1 *M* sodium citrate, 1.8 *M* (NH_4_)_2_SO_4_, 0.1 *M* Tris pH 8.5	10 mg ml^−1^ protein, 1.9 *M* (NH_4_)_2_SO_4_, 0.1 *M* sodium citrate pH 4.5
Final batch conditions (after mixing)	6.3 mg ml^−1^ protein, 1.4–1.6 *M* (NH_4_)_2_SO_4_, 48 m*M* citric acid, 53 m*M* Na_2_HPO_4_ pH 3.8	3–9 mg ml^−1^ protein, 2.2–3 *M* (NH_4_)_2_SO_4_ †, 75 m*M* Tris pH 8.3–8.5	3 mg ml^−1^ protein, 1.9 *M* (NH_4_)_2_SO_4_, 75 m*M* sodium citrate pH 4.5
Microcrystal size + shape	Adjustable between 5 and 60 µm diamonds	20 × 100 µm needles	15–20 µm tetrahedrons
Required for batch crystallization	Increase in [(NH_4_)_2_SO_4_]	Increase in [(NH_4_)_2_SO_4_]	Increase in [(NH_4_)_2_SO_4_]
		Omitting sodium citrate	
		Microseeding	
Improved crystallization	Exact [(NH_4_)_2_SO_4_]		Lower protein:precipitant ratio
	Humidity-controlled environment		Microseeding
	Removal of imidazole		

† There was no strong effect of ammonium sulfate concentration on crystal size in this range.

**Table 2:** Observations made for the batch micro-crystallization of AcNiR using different protein:precipitant ratios and thus different ammonium sulfate concentrations

Protein:precipitant ratio	1:1	1:2	1:3
Crystals	No	15–20 µm	15–20 µm
Precipitate	Light	Moderate	Heavy
Crystallization time	N/A	4–5 days (no seeds)	3–4 days (no seeds)
		1–2 days (seeds)	Overnight (seeds)

**Table 3:** Comparison of diffraction data from microcrystals (micro) and crystals obtained by vapour diffusion (macro) Data for microcrystals were collected using serial methods. Values in parentheses are for the outer shell. n.r., not reported.

	ADC		ECAO		AcNiR	
	Micro	Macro	Micro	Macro	Micro	Macro
Temperature (K)	293	100	293	100	298	298
No. of indexed frames/data sets	109708	1	15908	1	3018	1
Space group	*P*6_1_22	*P*6_1_22	*P*2_1_2_1_2_1_	*P*2_1_2_1_2_1_	*P*2_1_3	*P*2_1_3
*a*, *b*, *c* (Å)	72.8, 72.8, 219.0	72.2, 72.2, 216.1	134.7, 166.6, 79.8	135.1, 167.2, 79.9	96.0, 96.0, 96.0	96.2, 96.2, 96.2
α, β, γ (°)	90, 90, 120	90, 90, 120	90, 90, 90	90, 90, 90	90, 90, 90	90, 90, 90
Resolution range (Å)	63.1–2.0 (2.08–2.00)	23.6–2.2 (2.26–2.20)	104.8–2.5 (2.58–2.50)	20.0–2.0 (2.38–2.04)	96.0–2.0† (2.08–2.00)	48.1–1.41 (1.45–1.41)
〈*I*/σ(*I*)〉	19.3 (2.73)	6.4 (3.6)	3.47 (0.93)	7.9 (3.4)	2.59 (1.47)	5.1 (1.2)
Completeness (%)	100.0 (100.0)		100.0 (100.0)		100.0 (100.0)	
Multiplicity	6006 (4356)		67.2 (44.8)		43.1 (29.5)	
*R*_split_	0.04 (0.40)		0.27 (1.25)		0.33 (0.68)	
CC*	1.0 (0.95)	n.r.	0.99 (0.69)	n.r.		
CC_1/2_					0.95 (0.82)	0.99 (0.5)
Crystals used in	Monteiro *et al.* (2020 ▸)	Albert *et al.* (1998 ▸)	This work	Murray *et al.* (1999 ▸)	This work	Horrell *et al.* (2018 ▸)
PDB code	6rxh	1aw8		1dyu		5off

† The difference in resolution is a function of the relatively small SSX data set reported here. Using similar microcrystals, a SSX data set to 1.5 Å resolution was recorded and is reported in Ebrahim, Appleby *et al.* (2019 ▸).

## Footnotes

1 Data accessed through https://www.rcsb.org and https://www.ebi.ac.uk on 29 April 2020. Unique structures were determined by filtering for unique UniProt IDs (from PDBe). These entries were then searched for ammonium sulphate/sulfate and (NH_4_)_2_SO_4_ in the crystallization conditions (from RCSB PDB).
