# Supplementary material for: Homogeneous batch micro-crystallization of proteins from ammonium sulfate
Source: Acta Crystallogr D Struct Biol. 2021 Jan 26;77(Pt 2):194–204. doi: 10.1107/S2059798320015454 (PMC7869895; doi:10.1107/S2059798320015454)
Supplement: Supplementary file 1 [file d-77-00194-sup1.pdf]

STRUCTURAL BIOLOGY

Volume 77 (2021)

Supporting information for article:

Homogeneous batch micro-crystallization of proteins from ammonium sulfate

Claudia Stohrer, Sam Horrell, Susanne Meier, Marta Sans, David von Stetten, Michael Hough, Adrian Goldman, Diana C. F. Monteiro and Arwen R. Pearson

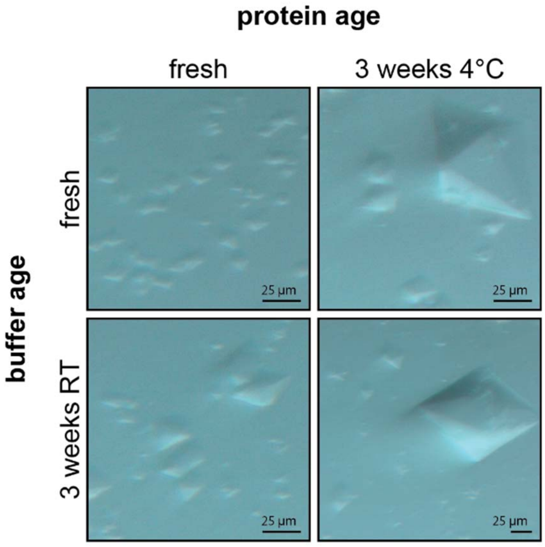

Figure S1 Effect of protein and buffer age for ADC crystallisation. Crystals were obtained by batch crystallisation using the same conditions (2 M $(NH_4)_2SO_4$) but either with freshly purified protein or protein stored at 4 °C for three weeks, and with buffer prepared from fresh stock solutions or buffer prepared from stock solutions which were stored at RT for three weeks. Both using old protein or old buffer leads to polydispersity in crystal size, and these effects are even more pronounced when combined.

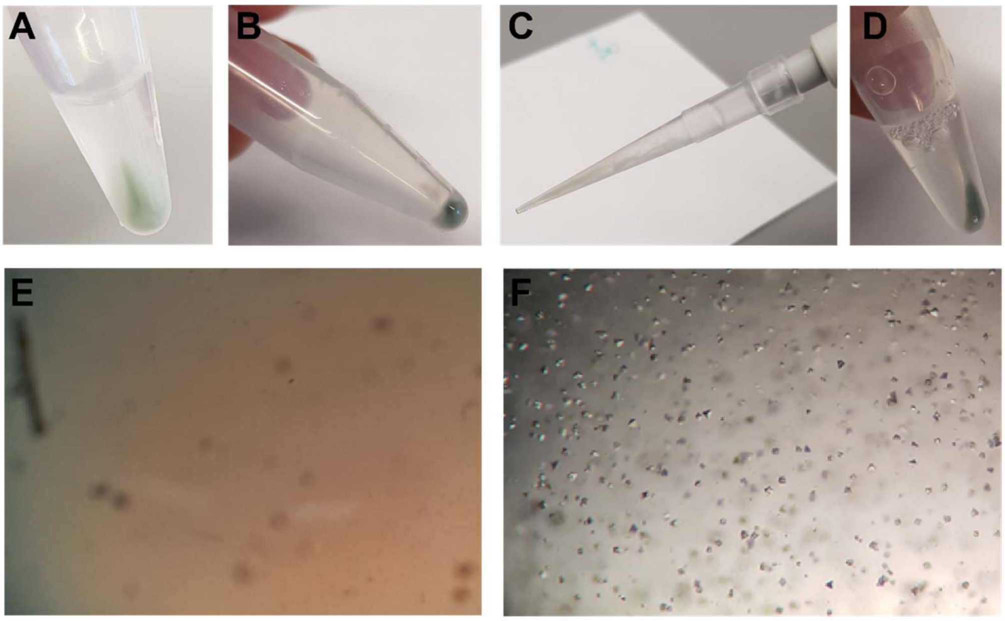

Figure S2 Separation of amorphous precipitant from AcNiR microcrystals. **A** AcNiR microcrystals and precipitant in Eppendorf tube after centrifugation at 2,300 x g for 1 minute. **B** crystals after removal of precipitate. **C** Removed precipitate in pipette. **D** AcNiR microcrystals and precipitant in Eppendorf tube after second centrifugation step. Less precipitant is visible. **E** Precipitate removed by centrifugation. **F** Crystals after removal of precipitate by centrifugation.
